# Pair-bond and survival in historical population: Marriage, widowhood, and social class

**DOI:** 10.1016/j.isci.2026.115759

**Published:** 2026-04-15

**Authors:** Jenni E. Pettay, Mirkka Lahdenperä, Antti O. Tanskanen, Virpi Lummaa, Mirkka Danielsbacka

**Affiliations:** 1 INVEST Research Flagship Centre, University of Turku, Turku, Finland; 2 Department of Biology, University of Turku, Turku, Finland; 3 Population Research Institute, Väestöliitto, Helsinki, Finland; 4 Department of Social Research, University of Turku, Turku, Finland

**Keywords:** social sciences, anthropology, history, interdisciplinary application studies

## Abstract

Marriage is widely associated with improved health and longevity, but spousal death significantly heightens immediate mortality risk, a phenomenon known as the “widowhood effect.” While these relationships are well-documented in modern populations, their prevalence in historical societies with extended family structures is less understood. Using church book records from Finland (1730–1910) and discrete-time event models, this study examined the survival probabilities of 11,892 individuals aged 40–90 across five marital statuses, with attention to socioeconomic status. In line with previous studies, we found large detrimental short-term widowhood effect. Long-term widowhood effect was smaller, and lower in women compared to men. Survival after remarriage was similar to first-time marriage. The survival of individuals who had never married did not differ from those who were married, suggesting that more communal living may have buffered against the possible negative effects of being single.

## Highlights

• Widowhood was associated with lower survival rates in both men and women
• The long-term widowhood effect was stronger in men
• Marital status showed a similar association with survival across socioeconomic classes
• The survival of those who were never married did not differ from that of married individuals

## Graphical abstract

**Figure:**
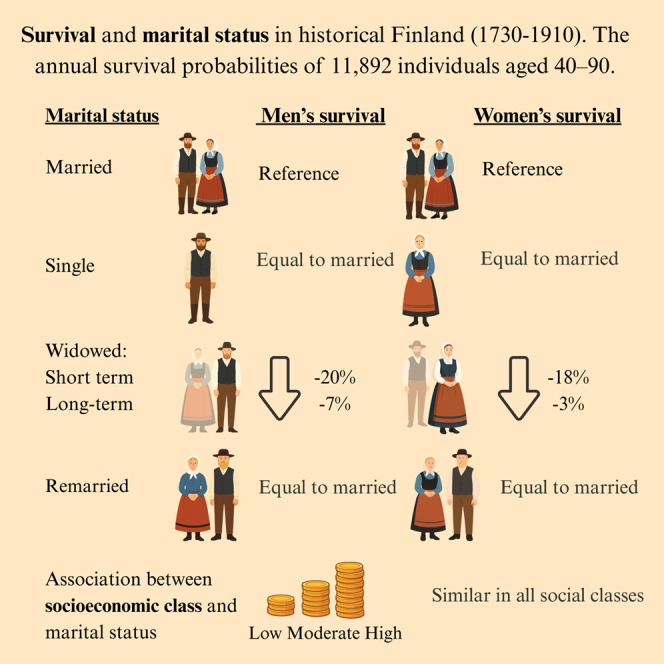

## Introduction

Long-lasting pair bonds are likely to be connected to the evolution of human life-history traits.^1^^,^^2^ For example, pair bonding and fathers’ long-term investment have supported the rearing of demanding offspring,^3^ and managing long-term pair-bonds might have contributed to the evolution of social intelligence and cooperative skills.^4^ Therefore, it is not surprising that we have evolved a desire for forming long-term pair-bonds, even in wealthy, highly individualistic societies.^5^

There is broad agreement that in contemporary populations, married individuals tend to experience lower mortality rates compared to those who are unmarried.^6^^,^^7^ The phenomenon of marital selection—where healthier individuals are more likely to marry and remain married—has been recognized for a long time as an important factor contributing to the better health and survival of married individuals.^8^ Longitudinal studies indeed suggest that persons with several health problems are more likely to become divorced.^9^ While this marriage selection effect partly explains the survival advantage of married individuals, there are additional theoretical mechanisms underlying the observed benefits of marriage.

First, economic advantages associated with marriage can vary across societies. For example, a country's pension system and women’s labor force participation affect the economic situation of widows.^10^ In the historical Finnish context studied here, economic advantages associated with marriage likely came mainly from living together and sharing household resources (e.g., risk sharing, division of labor, and economies of scale) rather than from institutional benefits tied specifically to legal marriage, such as joint taxation or survivor benefits, which were largely absent during the study period. Another suggested contribution for better health and longevity in marriage is health monitoring by partner, which can lead to a healthier lifestyle through, e.g., a healthier diet, physical activity, or not smoking.^11^ These associations can vary based on sex or socioeconomic status, e.g., health benefits from partner monitoring can be stronger in men^12^ and in lower educated people.^13^ Additionally, social support from a spouse has been linked to better mental health.^14^

Widowed individuals face a heightened risk of mortality. This “widowhood effect” is well-documented for both men and women across many societies.^15^^,^^16^ For example, Moon et al.^15^ (2011) conducted a meta-analysis across 12 contemporary populations, finding that the risk of death was elevated immediately following spousal loss (less than six months) with a 41% higher risk compared 14% higher risk during later widowhood. The same analysis suggested that the widowhood effect might be greater for men than women, although the sex difference in mortality was present only in certain populations. Similarly, a meta-analysis by Manzoli et al.^17^ using data from 53 studies of elderly individuals estimated mortality risks for widows and never-married individuals to be 11% higher compared to married individuals. Shor et al.^18^ analyzed 124 studies from different parts of the world and estimated the risk of death to be 27% higher for men and 15% higher for women.

The causes of increased mortality following spousal death are diverse, with cardiovascular diseases emerging as one of the most prominent,^19^ making the risk of dying from a “broken heart” more than just a metaphor.^20^ After the shock of bereavement, the risk of death is likely to be modified by the lack of the aforementioned health benefits associated with having a spouse. Notably, remarrying after bereavement is likely to restore marriage benefits and be associated with a similar mortality risk as a first-time marriage.^21^^,^^22^

Survival benefits of marriage are likely to be context-dependent. Marital status may have age-specific effects, with some studies finding stronger widowhood effects in younger age groups.^23^^,^^24^^,^^25^ Socioeconomic status is another important factor, as economically disadvantaged individuals in many populations are less likely to marry, contributing to higher mortality risks among singles. Additionally, individuals of higher socioeconomic status may mitigate the elevated mortality risks associated with being single. For example, in Thailand higher risk of mortality of single men was reduced after adjusting for income.^26^ Thus, it is essential to consider marital status and its interaction with socioeconomic indicators when studying mortality outcomes.

While the association between marital status and mortality is fairly well-studied in contemporary populations, it remains unclear whether similar patterns existed in the past when adult mortality rates were higher. To what extent are the observed benefits of being married influenced by modern factors such as access to medical care, individual dietary habits, and societal norms around living alone? For instance, some of the negative effects of being unpartnered or widowed may be relatively recent, as modern unpartnered individuals in wealthy societies often live alone.^27^^,^^28^ In the past, by contrast, unpartnered individuals were more likely to live with extended family, which might have resulted in a smaller survival difference between unpartnered and married. It is indeed suggested that the effects of widowhood on mortality are stronger in contemporary populations than in historical ones.^18^^,^^24^ While some explanations, such as economic support from marriage, likely applied in both historical and contemporary populations, others, such as health monitoring through medical visits, were likely less significant in historical contexts where healthcare was limited. By analyzing a population with markedly different sociopolitical and economic conditions from those of today, studies on pre-industrial populations can offer comparative insights into which aspects of the marriage-survival association may reflect fundamental social and economic mechanisms, and which are contingent on modern institutions and family structures.

To date, only a few studies have examined the relationship between marital status, bereavement, and mortality in historical populations. Nystedt,^29^ for example, analyzed 19th-century Swedish data on individuals aged 50–90 and found that widowhood had a stronger and more age-dependent effect on mortality in men than in women. Among men, short-term effects of widowhood weakened with age, while long-term effects strengthened. Poorer men were more affected, whereas no clear socioeconomic or age-related patterns were apparent for women. Gellently and Strömer^30^ used a representative data set from 19th-century France to investigate the relationship between mortality and being never married versus married, wealth at the time of death, and the age gap between spouses. They found that men and women who were married during their lives lived longer than never-married men and women, but this effect was stronger in men and was also seen in more advanced ages than in women. Wealthier people lived longer, and this was seen both in married and especially never married, but not among widows. Mineau et al. (2002) examined historical Mormon populations in Utah, analyzing cohorts married between 1860 and 1904, with mortality follow-up until 1990. They found that widowers had lower survival than widows and that widowhood at younger ages had stronger effects on men. Remarriage, larger family sizes, and religious involvement were linked to improved survival, indicating the central role of social support. Barclay et al. (2020) extended this research to polygamous families in historical Utah, finding that the death of a husband or sister wife increased mortality among women. For men, the death of one wife had a smaller impact if other wives survived. Interestingly, men and women in polygamous marriages had lower mortality than those in monogamous unions. These results highlight the importance of historical studies for understanding how marital and social relationships affect mortality in different contexts.

The present study examines individual-level data from Finnish church book records spanning 1730 to 1910. These records provide detailed information on all marriages, allowing us to account for all possible marital statuses: never married, married, widowed (both short- and long-term), and remarried. We follow individuals from age 40 throughout their lives to track time-varying changes in marital status. In this monogamous population, marriage was strongly encouraged, and divorce was extremely rare until the early 20th century. Adult mortality was relatively high, and many individuals experienced widowhood early in life. Extended families were common, with aging parents often living with their children or nearby.^24^^,^^31^^,^^32^ Unlike today’s Western societies, widows in historical Finland often lived with relatives, typically their children.^31^^,^^32^^,^^33^ Socioeconomic differences were large, leading to differences in the timing of reproduction, child number, and survival.^34^ Relatives typically provided care for impoverished individuals, while the poorest, known as parish paupers, were supported by rotating residence at different farms.^31^ Given these historical conditions, it can be predicted that poor, unmarried, and widowed individuals were particularly vulnerable. With these data, we are able to investigate how survival associated with marital status is shaped by age and socioeconomic status in a population with high mortality.

### Study aims

This study aims to determine whether the survival benefits of pair bonding observed in contemporary societies were also relevant in historical populations. Specifically, we address the following questions.

1. Was being married associated with higher survival compared to being never married or widowed?
2. Did widowhood result in a short-term increase in mortality risk, with less pronounced long-term effects?
3. Did men experience lower survival rates after spousal death compared to women?
4. Was remarriage associated with survival similar to that of first marriages?
5. Was the impact of widowhood on survival greater at younger ages than in later life?
6. Did lower SES amplify mortality risks for unmarried individuals, especially women?

## Results

### Men’s marital status and survival after age 40

We studied survival of men aged 40–90 in relation to their marital status categorized as: never married (single), married, newly widowed (maximum of 2 years after death bereavement), widowed, and remarried (Tables 1 and 2). First, we run model (M1) adjusting for marital status, age, quadratic age, socioeconomic status SES, region, and year. In the second model (M2), we added interaction between marital status and age, and marital status and SES, to explore potential age-specific effects and whether the relationship between survival and marital status varies according to socioeconomic status.

**Table 1:** Descriptive table of individuals

variable	Men		Women	
	N/mean	SD	N/mean	SD
sample size	5,739	–	6,153	–
no. of unknown death date	614	–	1,000	–
lifespan	63.67	12.65	66.35	13.10

**SES**				

wealthy	2,741	–	2,742	–
average	1,914	–	2,093	–
poor	1,084	–	1,318	–

**Region**				

Southwest Finland	1,933	–	2,129	–
Central Finland	1,983	–	2,104	–
Eastern Finland	672	–	638	–
Northern Finland	1,151	–	1,282	–

**Table 2:** Descriptive table of person-years of men and women

	men	women
	freq/median	freq/median
observations (N = person years)	114,037	135,020
deaths	3,980	4,009
age (median)	51	52
year (median)	1867	1869

**marriage status**		

single	4,398	6,574
married	73,531	70,402
newly widowed	4,168	5,861
widow	16,752	41,373
remarried	15,188	10,810

**SES**		

high	56,165	62,258
moderate	39,571	47,405
low	18,301	25,357

**Region**		

Southwest Finland	37,618	46,958
Central Finland	41,878	49,500
Eastern Finland	11,539	11,097
Northern Finland	23,002	27,465

We found a better fit in the model without interaction between marital status and age and marital status and SES based on AIC-value comparison (Table 3). Wald test for marital status and age also indicates the absence of age-specific effect (X^2^(4) = 5.72, *p* = 0.2). For interaction between marital status and SES, there is some support for men of moderate-SES to suffer more from short-term widowhood (X^2^(8) = 15.08, *p* = 0.06; Table 3, Figure 2). Furthermore, the model excluding interaction between age and marital status, but retaining SES interaction, has not a worse fit based on AIC value (32183.9), supporting cautious interpretation of lower survival of newly widowed in moderate-SES men.

**Figure 1:**
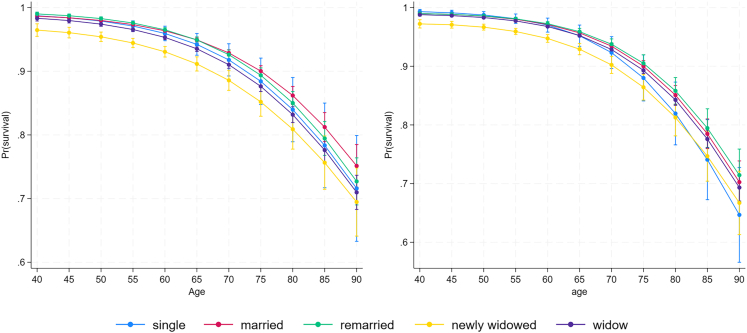
Marital status and survival Age-specific survival of (A) men and (B) women based on predictive margins and 95% confidence intervals from a complementary log-log regression model including interactions (M2) with different marital statuses (single, married, recently widowed, widow, and remarried).

Based on a more parsimonious model (M1), being recently widowed was associated with 20% lower survival compared to married men, whereas long-term widowhood was associated with 7% lower survival compared to being married (Table 1; Figure 1A). Survival of remarried individuals did not differ from that of men in first marriages according to the parsimonious model. Survival of never-married (single) men was similar to that of married men (Table 1; Figure 1A).

### Women’s marital status and survival after age 40

We studied survival of women aged 40–90 in relation to their marital status categorized as: never married (single), married, newly widowed (maximum of 2 years after death bereavement), widowed, and remarried (Tables 1 and 2). First, we run model (M1) adjusting for marital status, age, quadratic age, socioeconomic status SES, region, and year. In the second model (M2), we added interaction between marital status and age, and marital status and SES, to explore potential age-specific effects and whether the relationship between survival and marital status varies according to socioeconomic status.

**Figure 2:**
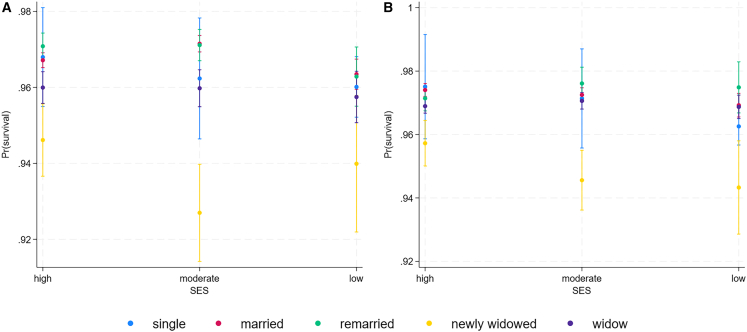
Survival based on marital status and SES Survival probabilities and 95% confidence intervals of the model including interaction terms (M2) based predictive margins (A) men and (B) women of different marital statuses and socioeconomic statuses (high, moderate, and low).

We found a better fit in the model without interaction between marital status and age and marital status and SES based on AIC-value comparison (Table 3). Wald test for marital status and age also indicates the absence of age-specific effects (X2(4) = 7.53, pp = 0.1), although results from M2 and interpretation from Figure 1A hint at a possible age-specific relationship between being single and age, being single having worse survival in older ages. Model retaining interaction between marital status and age and excluding SES-interaction has a similar AIC-value to the parsimonious M1 model (AIC = 33068.61). However, the possible effect size is very small, and the standard errors are large, indicating variation and a small sample size. Results do not indicate the presence of interaction between marital status and SES (X2(4) = 12.30, pp = 0.1; Table 3 and Figure 2B), suggesting the relationship between survival and marital status to be similar across socioeconomic classes.

**Table 3:** Results of event life history analysis of survival of men (*n* = 114,037 person years of 5,739 men) and women (*n* = 135,020 person years of 6,153 women)

Variable	Men M1			Men M2			Women M1			Women M2		
	AIC = 32183.32			AIC = 32186.11			AIC = 33068.33			AIC = 33071.65		
	HR	SE	P	HR	SE	P	HR	SE	P	HR	SE	P
age	**0.99**	**0.00**	**<0.0001**	**0.99**	**0.00**	**<0.0001**	**0.99**	**0.00**	**<0.0001**	**0.99**	**0.00**	**<0.0001**
Age quadratic	**1.00**	**0.00**	**<0.0001**	**1.00**	**0.00**	**<0.0001**	**1.00**	**0.00**	**<0.0001**	**1.00**	**0.00**	**<0.0001**

**marital status (married)**												

single	0.98	0.03	0.44	1.02	0.07	0.71	0.96	0.02	0.13	1.06	0.11	0.58
remarried	1.02	0.02	0.19	**1.06**	**0.02**	**0.01**	1.00	0.02	0.94	0.97	0.02	0.17
newly widowed	**0.80**	**0.02**	**<0.0001**	**0.84**	**0.03**	**<0.0001**	**0.82**	**0.02**	**<0.0001**	**0.83**	**0.03**	**<0.0001**
widow	**0.93**	**0.01**	**<0.0001**	**0.95**	**0.02**	**0.02**	**0.97**	**0.01**	**<0.01**	**0.94**	**0.02**	**<0.0001**

**SES (high)**												

moderate	1.02	0.01	0.086	**1.04**	**0.02**	**0.003**	1.00	0.01	0.88	0.98	0.02	0.27
low	**0.96**	**0.01**	**0.012**	0.97	0.02	0.09	**0.97**	**0.01**	**0.03**	**0.95**	**0.02**	**0.01**

**marital status ∗ age**												

single	–	–	–	1.00	0.00	0.43	–	–	–	**1.00**	**0.00**	**0.04**
remarried	–	–	–	1.00	0.00	0.06	–	–	–	1.00	0.00	0.71
newly widowed	–	–	–	1.00	0.00	0.37	–	–	–	1.00	0.00	0.14
widowed	–	–	–	1.00	0.00	0.35	–	–	–	1.00	0.00	0.82

**marital status∗SES**												

single∗moderate	–	–	–	0.91	0.09	0.31	–	–	–	0.97	0.13	0.84
single∗low	–	–	–	0.96	0.07	0.61	–	–	–	0.92	0.10	0.45
remarried∗moderate	–	–	–	0.96	0.03	0.21	–	–	–	1.07	0.05	0.09
remarried∗low	–	–	–	0.96	0.04	0.32	–	–	–	1.10	0.06	0.11
newwidow∗moderate	–	–	–	**0.85**	**0.04**	**<0.0001**	–	–	–	0.94	0.04	0.15
newly widow∗low	–	–	–	0.99	0.07	0.93	–	–	–	0.95	0.06	0.44
widow∗moderate	–	–	–	0.96	0.03	0.12	–	–	–	1.04	0.02	0.13
widow∗low	–	–	–	1.01	0.04	0.72	–	–	–	1.05	0.03	0.10

**Region (Southwest Finland)**												

Central Finland	**1.05**	**0.01**	**<0.0001**	**1.05**	**0.01**	**<0.0001**	1.02	0.01	0.08	1.02	0.01	0.09
Eastern Finland	0.97	0.02	0.12	0.97	0.02	0.12	**0.92**	**0.02**	**<0.0001**	**0.91**	**0.02**	**<0.0001**
Northern Finland	1.01	0.01	0.31	1.01	0.01	0.31	**0.97**	**0.01**	**0.03**	**0.97**	**0.01**	**0.02**
year	**1.00**	**0.00**	**<0.0001**	**1.00**	**0.00**	**<0.0001**	**1.00**	**0.00**	**0.0000**	**1.00**	**0.00**	**<0.0001**

Second model (M2) included interaction terms between marital status and age and socioeconomic status (SES).

We used complementary log-log regression with annual survival (survived = 1, death = 0) as the dependent variable. The time-varying predictors used in the models were: age, marital status, and year. Time-invariant predictors were socioeconomic status (SES) and region. Significant effects are indicated in bold (α = 0.05), SE standard error. In men, marital status∗age -interaction was removed from the final model based on a smaller AIC. The reference category is shown in brackets.

Based on the parsimonious model (M1), being a recently widowed woman had 18% lower survival compared to married women, whereas long-term widowhood was associated with 3% lower survival compared to being married (Table 1; Figure 1A). Survival of remarried women did not differ from that of women in first marriages. Survival of never-married (single) women was similar to that of married women (Table 1; Figure 1A).

### Sex differences

**Figure 3:**
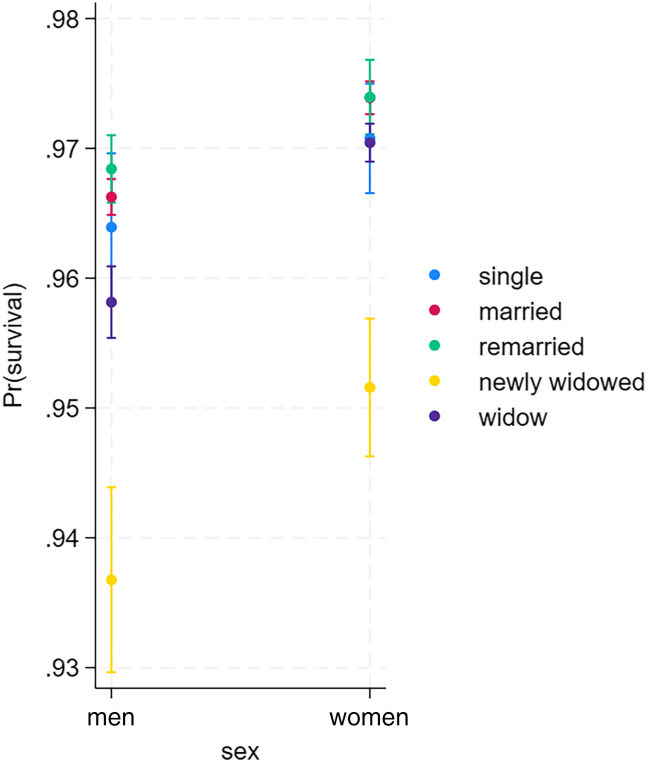
Survival of men and women of different marital statuses (single, married, recently widowed, widow, and remarried) based on predictive margins and 95% confidence intervals from a complementary log-log regression model

We run analyses including men and women to test sex differences in survival according to marital status. Results suggest that while survival of never married, married, remarried, and newly widowed were similar in both sexes, women suffered less from long-term widowhood (Table 4; Figure 3).

**Table 4:** Results of event life history analysis of survival of men and women to test sex differences in survival according to the marital status (*N* = 249,057)

Variable	HR	SE	P
age	1.03	0.00	<0.0001
Age quadratic	1.00	0.00	<0.0001

**sex (man)**			

woman	1.08	0.01	<0.0001

**marital status (married)**			

single	0.98	0.03	0.43
remarried	1.02	0.02	0.16
newly widowed	0.80	0.02	<0.0001
widow	0.93	0.01	<0.0001

**SES (high)**			

moderate	1.01	0.01	0.29
low	0.97	0.01	0.001

**sex∗marital status**			

woman∗single	0.99	0.03	0.71
woman∗remarried	0.98	0.02	0.38
woman∗newly widowed	1.02	0.03	0.55
woman∗widowed	1.03	0.02	0.047

**Region (Southwest Finland)**			

Central Finland	1.03	0.01	<0.0001
Eastern Finland	0.94	0.01	<0.0001
Northern Finland	0.99	0.01	0.38
year	1.00	0.00	<0.0001

### Sensitivity analysis

While the average marriage age in this population was in mid-twenties, some people married at older ages; here, 34% of men and 36% women who were single at 40 married later in their lives. Those who married late might differ from those who never married. As a sensitivity analysis, we investigated whether those men and women who did not marry during their lifetime were different from those who married later in life. We restricted the sample to include only those singles who never married during their lifetime, vs. those who were married, excluding widowhood and remarriage life-stages. This much restricted sample suggests that the survival of those men who never married during their lifetime (NN = 216) had 5% lower survival than married men, but this result was only marginally statistically significant (H±SE: 0.95 ± 0.03, p = 0.07). Similarly, women who never married (307) had 5%, but not statistically significant (H±SE: 0.95 ± 0.03, *p* = 0.10) lower survival than married women. See Table S1 for full models.

## Discussion

In this study, we used historical data from 18th- and 19th-century Finland to investigate how marital status relates to survival. Historical populations offer a valuable comparison to contemporary populations due to their higher mortality rates, lack of effective medical care, and more communal living conditions. We were able to track individuals from age 40 until death, examining survival of those who were never married (single), married, widowed, or remarried. Importantly, our dataset allowed us to account for socioeconomic class based on landownership and occupation, which serve as indicators of social class in this otherwise relatively homogenous population.

We first hypothesized that being married would be associated with higher survival compared to being single or widowed, consistent with findings from previous studies in other populations. Here, this was generally supported, as being married was associated with better survival than being widowed.

Our second question was whether widowhood results in a more pronounced short-term increase in mortality risk with less pronounced long-term effects. We indeed found a clear “shock effect” following bereavement in both sexes, suggesting the universality of this phenomenon. Higher mortality after the death of a spouse has been well-documented in many studies. For example, in late 20th-century Finland, Martikainen & Valtonen^23^ examined records of over 1.5 million individuals to assess mortality following spousal loss.^23^ They observed that mortality increased by 29% in men and 22% in women within the first six months after bereavement, and by 19% in men and 7% in women beyond the six-month period following bereavement. Our estimates for short-term widowhood effect were 20% for men and 18% for women, and for long term 7% and 3%, respectively. The model combining sexes suggests that while otherwise sexes show similar patterns, the long-term widowhood effect was smaller in women compared to men. This finding provides partial support for our third hypothesis that men might experience a stronger widowhood effect than women, as has been suggested in studies of contemporary populations.^35^ However, a large meta-analysis^17^ suggests that the overall effects of marital status on survival are similar for men and women, though variation in individual studies highlights the influence of environmental and societal factors on sex differences in these associations.

One possible factor contributing to increased mortality among surviving spouses could be shared environmental risks. For example, spouses might have succumbed to the same infectious disease, particularly in the absence of effective medical care. Unfortunately, this possibility is difficult to fully explore in our dataset. Although parish vicars recorded causes of death, these records are more reliable for childhood deaths due to infectious diseases,^36^^,^^37^ while causes for older adults were often vague, with terms such as “old age” or “wasting” being commonly reported. This limitation makes it challenging to assess the causes of death that contribute to the observed trends in lower survival associated with widowhood.

Our fourth question was whether remarriage was associated with survival similar to that of first marriages. In this population, remarriage was only possible after the death of a spouse. Regardless of social class, remarried individuals had survival rates similar to those of first-time married individuals. These findings indeed suggest that remarriage could provide comparable survival benefits to those of first marriages in both men and women across all social classes. However, remarried individuals are probably selected for their health, wealth, or other attributes associated with survival, it is impossible to say how much comparable survival is due to selection or lifestyle benefits brought by marriage.

Some previous studies have reported that the widowhood effect is stronger in earlier life than in later life.^23^^,^^24^^,^^25^ Which led us to question if the impact of widowhood on survival is greater at younger ages than in later life. However, we did not observe any notable age effect on marital status and survival. In this historical population, widowhood was more common at younger ages compared to contemporary populations. For instance, by age 50, almost 30% of individuals in our sample had already experienced the death of a spouse.

We were interested in whether lower SES amplifies mortality risks for unmarried individuals, especially women. Our results suggest the association of survival and marital status was similar across social classes in this population. However, low-SES men and women had overall lower survival compared to high-SES men and women.

Most contemporary studies suggest that never being married would be associated with lower survival and health outcomes than being married.^38^ Additionally, limited historical data indicate that never-married individuals tended to have shorter lifespans.^30^ However, our findings revealed that single men and women had comparable survival rates to their married counterparts. One reason why never-married individuals were not suffering from lower survival might be because in this agrarian society, people seldom lived alone, whether they were married, unmarried, or widowed.^39^ Some aspects of historical marriage might be a reason why there is less survival gap between being single and being married. First, as farming was typically managed by married couples, unmarried individuals were not burdened with the responsibility of running a farm and supporting children and other household members. Second, as divorce was absent, people had to stay in unhappy, unsupportive marriages, which might reduce the gap between being married and single.

Furthermore, individuals who married later in life might differ from those who remained unmarried for their whole life; over a third of those who were single at 40 married later in life. Our sensitivity analysis that restricted analysis for those unmarried for life showed ∼5% lower survival than married, but these estimates were not statistically significant. While this result should be interpreted with caution because of the reduced sample size, one might expect those who never marry might suffer health conditions, which select them out from marriage in this society.

Our study has several strengths. We examined survival in relation to marital status in all its possible forms within this population: never married, married, widowed, and remarried. Importantly, we distinguished between the immediate widowhood period following spousal death and long-term widowhood, recognizing that the shock of bereavement differs from other potential challenges associated with widowhood. Moreover, our sample does not suffer from biases related to institutionalized individuals, which can affect contemporary settings.^17^ A key contribution of this study is its focus on how survival varies with marital status across socioeconomic classes, rather than merely controlling for socioeconomic factors. We also observed a strong widowhood effect immediately after bereavement, but the long-term survival associations were less severe than many estimates from modern populations. Nevertheless, we found a more consistent long-term survival disadvantage in men compared to women, aligning with some contemporary studies. Comparisons of exact effect sizes remain challenging, as many studies do not separate immediate and long-term widowhood effects and often use varying age groups and covariates in their analyses. However, our estimates for widowhood effects are smaller compared to some of the contemporary studies, and this aligns with findings from other historical populations.^18^^,^^24^^,^^40^^,^^41^ Interestingly, we did not find support that being single would have been clearly detrimental for survival, as in many contemporary populations. While living in 18th- and 19th-century Finland was harsh in many ways, resulting in high mortality, it is possible that communities played a supportive role for bereaved or unmarried individuals. Further studies exploring how pair bonding influences survival—while considering factors such as socioeconomic status, living arrangements, and reproduction—could provide deeper insight into the mechanisms driving survival differences based on marital status. Such investigations provide valuable comparisons to modern populations and contribute new perspectives on the relationship between survival and marriage by highlighting the importance of historical context. In our historical Finland, not being married was not associated with living alone, like in most contemporary populations, which might mean more moderate effects of marriage. Although changes in family structure may influence the protective effects of marriage, our data do not allow the direct measurement of household composition. Nevertheless, it is well-known that living alone was uncommon during the study period,^39^ and widowed individuals likely resided with relatives, suggesting that marital status effects may have operated within broader kin-based support systems. However, the lack of individual-level data on family structure remains an important limitation.

### Limitations of the study

Our study has some limitations. One clear limitation is the correlational nature of the data, which prevents us from establishing causality between marital status and mortality. The observed associations may be influenced by confounding factors, such as shared environments between spouses, which could partly explain the mortality associated with widowhood. Another limitation is the aforementioned lack of information on living arrangements. We do not have data on whether never-married or widowed individuals lived alone or with others, which limits our ability to fully interpret the survival patterns observed in these groups. Understanding specific living arrangements could provide valuable insights, particularly when examining how socioeconomic class interacts with marital status to influence survival outcomes. Lastly, while our socioeconomic classification is reasonably robust, it is likely that variations exist within the three broad socioeconomic classes used in this study. For example, the low social class includes individuals who could have been in stable service employment and parish paupers who did not have any economic security. A more detailed categorization of socioeconomic strata could help refine our understanding of the relationship between marital status, class, and survival.

## Resource availability

### Lead contact

Requests for further information and resources should be directed to and will be fulfilled by the lead contact, Jenni Pettay (jenni.pettay@utu.fi).

### Materials availability

This study did not generate new unique materials.

### Data and code availability

• Data have been deposited in Mendeley Data: https://data.mendeley.com/datasets/3x5nfy5wvz/1 and are publicly available as of the date of publication.
• Data and Stata code have been deposited in Mendeley Data: https://data.mendeley.com/datasets/3x5nfy5wvz/1.
• Any additional information required to reanalyze the data reported in this paper is available from the lead contact upon request.

## Acknowledgments

The study is funded by the Strategic Research Council established within the 10.13039/501100002341Research Council of Finland to the NetResilience consortium (grant nos. 364371 and 364385) and the INVEST Research Flagship (grant no. 345546). We also acknowledge funding from the 10.13039/501100002341Research Council of Finland (ML, grant no. 371390; AOT, grant no. 338869), 10.13039/501100000781European Research Council to KinSocieties (V.L., ERC-2022-ADG, grant no. 101098266), Human Diversity consortium, under the Profi7 program (VL, ML, grant no. 352727) and the Centre of Excellence (grant no. 374221) by the Research Council of Finland. We thank all the genealogists and research assistants involved in collecting and digitizing parish records for this study, particularly Kimmo Pokkinen and Aino Siitonen. We also want to thank anonymous reviewers for helpful comments.

## Author contributions

Conceptualization, J.P., M.D., A.O.T., M.L., and V.L.; data curation, J.P. and M.L.; formal analysis, J.P.; funding acquisition, A.O.T., M.D., and V.L.; investigation, J.P.; methodology, J.P.; project administration, M.D., A.O.T., and V.L.; supervision, M.D.; visualization, J.P.; writing – original draft, J.P.; writing – review and editing, J.P., M.D., M.L., and A.O.T.

## Declaration of interests

The authors declare no competing interests.

## STAR★Methods

### Key resources table

**Table:** 

REAGENT or RESOURCE	SOURCE	IDENTIFIER
Data used in this study	Pettay et al.^42^	https://data.mendeley.com/datasets/3x5nfy5wvz/1

**Software and algorithms**		

Code used in this study	Stata code	https://data.mendeley.com/datasets/3x5nfy5wvz/1
Stata 19.0	StataCorp^43^	

### Experimental model and study participant details

Omitted as our study does not involve biological models.

### Method details

#### Data

We use an extensive pre-industrial demographic dataset collected from population registers of historical Finland.^44^ These registers detailing births, deaths, children, occupations, and marriages were maintained by the Lutheran Church, and were used to construct full life-histories of individuals.

Finnish society in the eighteenth and nineteenth centuries was agrarian and largely pre-dates industrialisation without effective health care. Overall, marriage was seen as desired/natural state of living and running of a farm needed labor of more than one adult. First age of marriage was (mean±stderr) 27.45±6.17 for men and 25.93±6.48 for women in this sample. Legal divorce was extremely rare before 1910’s.^45^ Over 90% people that survived to age 45 married during their lifetime, but lower socioeconomic status was associated with lower probability of marrying.^46^ Because of relatively high adult mortality, widowhood could happen in relatively young ages. Age at first time widowhood was on average 61.14±18.41 for men and 56.11±16.01 for women in our sample. Men were more likely to remarry than women, and younger age was related to higher probability remarry after widowhood, especially for women.^47^^,^^48^^,^^49^^,^^50^ In our sample 18% men and 12% of women remarried during their lifetime. However, only 2,6% men and 0.8% women were married more than twice in their lifetime.

We categorised individuals as belonging to one of the three following social classes, which can capture important between-individual variation in mortality, marriage patterns, and birth rate: high (mainly farm owners), moderate (tenant farmers, fishermen, craftsmen), and low (servants, cottagers) (see more details Salonen et al., 2024). SES was estimated at the family formation ages around 30’s, so SES is time-invariant, although many young people served in different farms before establishing their own farm and household after marrying. Because SES is so important in this population,^34^ we only included individuals with known social class, while SES was missing for ∼5 % persons from original sample. Death percentages of missing-SES was close to average death percentage across all known social classes (3.2% versus 3.1%). We also included persons with known birth region, which was missing for ∼ 4 %. Survival in missing category was 3.7% versus 3.2 % of average of known regions. Region was included to take into account variation in mortality due to regional differences, parishes were combined to four ecologically and socially distinct areas: Southwest Finland, Central Finland, Eastern Finland, and Northern Finland.

### Quantification and statistical analysis

Our sample included 5,739 men and 6,153 women (Table 1.). First, we analysed sexes separately, because of moderate lifespan differences of men and women (here: 64 and 66, respectively), and then we analysed sexes together to test whether survival patterns differ between the sexes. Survival was analysed with discrete time-event complementary log-log regression,^51^^,^^52^ which allowed us to analyse the effects of time-dependent variables on a yearly basis. Complementary log-log regression is especially suited when events are rare and is often suitable for survival analysis as the regression coefficients are identical to those of an underlying proportional hazards regression model.^52^^,^^53^ When death was unknown, or death took place after year 1910, individuals were followed until their last record in the data or year 1910. Sample for men was 114,039 and for women 135,020 observations, description of observation data is presented in Table 2. Each year from age 40 to death (or until follow-up in case of individuals of unknown death date) of individual’s life was treated as an observation. Starting age was chosen to be 40, because before that data included relatively few widowhoods and remarriages, and we also removed ages after 90, because number of observations were very few after that age in this population. Our response variable was annual survival (1 = surviving and 0 = death in that year). Our main interest was marriage status of individuals, which could change several times during lifetime and our follow-up. Marital status “married” was used as a reference category. Marital stages were called: never married (single) before the first marriage, married, recently widowed, widow, and remarried containing all marriages after the first marriage. Single here refers to state of never being married, e.g. time before marriage. Change of marriage status for married and remarried were coded for following year that (re)marriage took place. To differentiate shock effect of widowhood (“widow effect”) and widowhood as life stage we created two levels of widowhood; recently widowed person years included year when spouse died and following year, as a robust time span for immediate shock effect affecting mortality would happen being around 1 year.^16^ Second widow-status includes all person years after the second year after spouse death until remarriage, death or censoring. Widowhood and remarriage could have happened more than one time, and we coded these statuses as widowed and remarried not distinguishing between first and second widowhood or remarriage due to relatively small sample size. This data was collected as a pedigree collecting life histories of descendants of starting generation individuals, which leads to some limitations. While we have information for individuals who married “pedigree” person, they are in our data because they survived until marriage. Therefore, we only included observation years from marriage onwards for those individuals who entered data by marrying “pedigree” person. This leads to relatively smaller sample for single-category. When spouses’ death year from first or second marriage was missing (32%), we dropped those marriage years from data, because timing of possible widowhood could not have been determined. Running analysis with unknown marital status observations as one category did not change our results, and survival in unknown category was similar to survival of married (Table S2).

Age was included in the model as linear and quadratic term. Age was centered to median 51 for men and 52 for women for easier interpretation. Interaction with age and marriage status was included, because marriage status can relate with survival differently in different ages. Social class was added as three level class-variable (high, moderate, and low). Region was included to take into account variation in mortality due to regional differences (4 levels: Southwest Finland, Central Finland, Eastern Finland, and Northern Finland). Year was included as continuous variable, because of general trend of increase in lifespan from 1730 to 1910.

We included robust standard errors. To interpret and visualize results, especially age specific survival with marital status, and between social class and marital status, we calculated predictive margins from the regression models.^54^ All statistical analyses were performed using Stata 18 (StataCorp, 2023).
