# Supplementary material for: Pair-bond and survival in historical population: Marriage, widowhood, and social class
Source: iScience. 2026 Apr 15;29(5):115759. doi: 10.1016/j.isci.2026.115759 (PMC13156997; doi:10.1016/j.isci.2026.115759)
Supplement: Document S1. Tables S1 and S2 [file mmc1.pdf]

iScience, Volume *29*

Supplemental information

Pair-bond and survival in historical population: Marriage, widowhood, and social class

Jenni E. Pettay, Mirkka Lahdenperä, Antti O. Tanskanen, Virpi Lummaa, and Mirkka Danielsbacka

Supplementary materials

Supplementary table 1. Results of event life history analysis of survival of men and women who never married versus those men and women who were married (reference category).

	**Men** N=77,237			**Women** N=76,221			
survival	HR	SE	P	HR	SE	z	P
age	0.99	0.00	<.0001	0.99	0.00	-16.08	<.0001
Age quadratic	1.00	0.00	<.0001	1.00	0.00	-6.08	<.0001
marital status (married)							
single	0.95	0.03	0.07	0.95	0.03	-1.67	0.10
SES (high)							
moderate	1.04	0.02	0.005	0.99	0.02	-0.77	0.44
low	0.97	0.02	0.16	0.96	0.02	-2.00	0.05
Region (Southwest Finland)							
Central Finland	1.05	0.02	<.0001	1.03	0.02	2.02	0.04
Eastern Finland	0.96	0.02	0.09	0.94	0.03	-2.24	0.03
Northern Finland	1.01	0.02	0.54	0.97	0.02	-1.76	0.08
year	1.00	0.00	<.0001	1.00	0.00	4.13	<.0001

Supplementary table 2. When spouses' death year from first or second marriage was missing, we focal persons marital status could not be determined. Death year of first spouse was missing for 32%. To see if dropping these missing observations would bias our results, we run sensitivity analysis that included these unknown marital statuses by assigning them to category "unknown". Running analysis with unknown marital status observation years as one category did not change our results.

	Men (N=155,047)			Women (N=168,530)		
survival	HR	SE	P	HR	SE	P
age	1.02	0.00	<.0001	1.04	0.00	<.0001
Age quadratic	1.00	0.00	<.0001	1.00	0.00	<.0001
marital status (married)						
single	0.96	0.03	0.19	0.96	0.02	0.08
remarried	1.02	0.02	0.17	1.00	0.02	0.99
Unknown	1.01	0.01	0.24	1.01	0.01	0.60
newly widowed	0.80	0.02	<.0001	0.82	0.02	<.0001
widow	0.93	0.01	<.0001	0.96	0.01	<.001
SES (High)						
Moderate	1.02	0.01	0.07	1.00	0.01	0.91
Low	0.98	0.01	0.213	0.97	0.01	0.04
Region (Southwest Finland)						
Central Finland	1.05	0.01	<.0001	1.02	0.01	0.02
Eastern Finland	0.97	0.02	0.09	0.92	0.02	<.0001
Northern Finland	1.01	0.01	0.37	0.98	0.01	0.05
year	1.00	0.00	<.0001	1.00	0.00	<.0001
